# New Appliances and Things Medical

**Published:** 1911-02-04

**Authors:** 


### NEW APPLIANCES & THINGS MEDICAL.

[We shall be glad to receive at our Office, 28 & 29 Southampton Street, Strand, London, W.C., from the manufacturers, specimens of all new preparations and appliances.]

A PROPHYLACTIC AGAINST SEA-SICKNESS.

(Validol Agency, 33 Lime Street, E.C.)

We have not been able to test the efficacy of this combination on ourselves, but tests carried out on patients who have been martyrs to a "liability to sea-sickness" show that the claims made on behalf of the remedy are not exaggerated. Validol is a combination of menthol and valerianic acid—chemically a valerianate of menthol—of an agreeable aromatic odour, and with hardly any appreciable taste. It is easily prescibed by dropping the liquid on lumps of sugar, which are taken by the patient just before or immediately on arriving on board ship. The remedy seems to relieve patients considerably, and in most cases is a trustworthy prophylactic, though in the severe types of sea-sickness in which there is persistent vomiting, accompanied with collapse, "Validol camphoratum" is said to be more efficacious. Validol is well worth a trial by everyone who has a dread of sea-sickness, or who suffers when crossing the Channel.

